# Research on the Comfort of Vehicle Passengers Considering the Vehicle Motion State and Passenger Physiological Characteristics: Improving the Passenger Comfort of Autonomous Vehicles

**DOI:** 10.3390/ijerph17186821

**Published:** 2020-09-18

**Authors:** Chang Wang, Xia Zhao, Rui Fu, Zhen Li

**Affiliations:** School of Automobile, Chang’an University, Xi’an 710064, China; wangchang@chd.edu.cn (C.W.); zhaoxia26@chd.edu.cn (X.Z.); lizhen@chd.edu.cn (Z.L.)

**Keywords:** autonomous vehicle, vehicle motion state, motion sickness, physiological characteristic, passenger comfort, BiLSTM

## Abstract

Comfort is a significant factor that affects passengers’ choice of autonomous vehicles. The comfort of an autonomous vehicle is largely determined by its control algorithm. Therefore, if the comfort of passengers can be predicted based on factors that affect comfort and the control algorithm can be adjusted, it can be beneficial to improve the comfort of autonomous vehicles. In view of this, in the present study, a human-driven experiment was carried out to simulate the typical driving state of a future autonomous vehicle. In the experiment, vehicle motion parameters and the comfort evaluation results of passengers with different physiological characteristics were collected. A single-factor analysis method and binary logistic regression analysis model were used to determine the factors that affect the evaluation results of passenger comfort. A passenger comfort prediction model was established based on the bidirectional long short-term memory network model. The results demonstrate that the accuracy of the passenger comfort prediction model reached 84%, which can provide a theoretical basis for the adjustment of the control algorithm and path trajectory of autonomous vehicles.

## 1. Introduction

### 1.1. Background

Research on autonomous vehicles is in full swing. Autonomous vehicles are emerging primarily to provide travel services for humans, and the ride comfort of cars is an important evaluation criterion that reflects travel services. In addition, there is a strong correlation between the comfort and acceptance of autonomous vehicles [1]. However, according to an new international survey on carsickness by Eike et.al., they surveyed 4479 people from China, the UK, Brazil, and Germany about their experiences of carsickness and found that 46% of them had experienced some degree of carsickness within five years [2]. They also pointed out that the country with the highest rate (61.7%) of motion sickness is China.

Comfort is a subjective feeling, and there is no unified and clear definition for it in academia. The controversy surrounding comfort is focused on the understanding of the difference between comfort and discomfort. Some scholars have divided comfort into two discrete states: namely, the presence of comfort and the lack of comfort. Thus, comfort is simply defined as the absence of discomfort [3]. The improvement of passenger comfort discussed in the present article primarily refers to avoiding the discomfort of passengers during a car ride as much as possible, and motion sickness is a serious type of discomfort.

Existing research on comfort in traditional vehicles has mainly concentrated on the driving behavior of the driver, the design of the vehicle chassis, and the interior environment of the vehicle [4,5,6]. Compared with traditional vehicles, in highly autonomous vehicles, the driver transforms form a vehicle controller to a passenger capable of performing non-driving tasks, which may bring new challenges to passenger comfort. Schoettle and Sivak [7] conducted research on drivers and passengers in five countries: the United States, China, the United Kingdom, Japan, and India. They considered possible non-driving activities (such as chatting with friends, watching TV, etc.) and made predictions on the comfort of the passengers; the results showed that 6%–10% of Chinese passengers are expected to experience varying degrees of motion sickness in fully autonomous vehicles, and that 6%–13% will sometimes experience moderate or severe motion sickness symptoms in fully autonomous vehicles, as compared with 4%–7% and 4%–9% in the UK, respectively.

With the development of control algorithms, the pursuit of intelligent driving vehicles is no longer limited to safety, and the improvement of comfort is inevitable. An uncomfortable riding experience will reduce people’s acceptance of autonomous vehicles. However, if we can understand the factors affecting passengers’ discomfort, and accordingly improve the intelligent driving control strategy and optimize the interaction between intelligent driving vehicles and passengers, the frequency and degree of carsickness of passengers will be effectively reduced, and the riding comfort of passengers will be improved.

### 1.2. Research Status

#### 1.2.1. Research Method of Relationship between Vehicle Motion Characteristics and Passenger Comfort

As early as 1976, on the basis of summarizing a large number of studies on passenger comfort, Hoberock [8] pointed out that the main method to study passenger comfort is to let passengers experience different driving conditions in vehicles or special equipment. Through questionnaire survey, subjects were asked to make subjective evaluation on riding comfort, and finally establish the relationship between passengers’ subjective comfort and vehicle motion parameters. However, the questionnaire on passenger comfort is only conducted once at the end of each journey, so the overall comfort level is obtained through the questionnaire [4,9,10]. As a result, the passenger comfort score has no temporal relationship with the operation of a single vehicle, and the relationship between passenger comfort and discrete vehicle movement cannot be determined. Subsequently, some researchers proposed digital comfort scoring methods, such as the fast motion sickness scale (FMS) based on verbal rating [11]. Passengers were required to rate the comfort once per minute, with a score range of 0 (no sickness at all) to 20 (frank sickness). The FMS provides a possibility to record passenger comfort during vehicle motion operations. However, it has its own limitations. For example, the FMS cannot collect the comfort of passengers in the operation of the vehicle at a certain moment. 

The traditional test method is limited by the passengers not being able to evaluate the comfort in real time, which leads to the inability to accurately analyze the impact of vehicle motion parameters on passenger comfort. In our experiment, the subjects can evaluate the comfort in real time, combined with vehicle data, which can accurately analyze the relationship between vehicle motion parameters and passenger comfort.

#### 1.2.2. Research on the Relationship between Passenger Physiological Characteristics and Ride Comfort

Some researchers took the physiological characteristics of passengers as an objective measurement method of comfort. They took the changes of physiological parameters as the representation of changes in passenger comfort and analyzed a series of physiological parameters related to passenger comfort changes, such heart rate [12,13,14,15], blood pressure [16,17], and skin conductance level [12]. Some researchers adopted subjective measurement methods to study passenger comfort, allowing passengers to evaluate comfort based on their own feelings. In subjective measurement methods, the physiological characteristics of passengers were often used as factors affecting passenger comfort. Powell and Palacín [18] summarized the influences of acceleration on balance and passengers’ tolerable range of acceleration from physiological and kinematic perspectives and considered that different individuals have varied tolerances for longitudinal acceleration due to differences in physiological and psychological mechanisms. This conclusion was conducive to the setting of vehicle acceleration threshold, but this study did not point out the specific physiological characteristics that cause the difference of passenger’s acceptance of vehicle motion parameters. Koslucher et al. [19] evaluated the gender differences in motion sickness induced by linear visual oscillation, and evaluated the incidence of motion sickness and the severity of related symptoms. It was found that the incidence rate of motion sickness in women was 38%, while that in men was only 9%. Therefore, it was proposed that the riding comfort of passengers of different genders was different, and that these differences varied under different vehicle motion stimuli. Based on the comfort experience of aircraft passengers, Ahmadpour et al. [20] studied the effects of eight different factors, such as peace of mind, physical health, and pleasure, on the comfort experience of the passengers and found that some factors of the physiological state of passengers themselves have certain influences on their flight comfort experience. Based on the above research, and considering the different factors of carsickness in different environments, we used a subjective measurement method to analyze the comprehensive influence of vehicle operating parameters and passenger physiological characteristics on passenger comfort under different vehicle motion conditions, considered the difference of different passengers’ acceptance of vehicle operating parameters, and then established a comfort prediction model considering passenger physiological characteristics.

#### 1.2.3. Intelligent Driving Technology Considering Passenger Comfort

Many existing studies have designed a variety of control strategies for different scenarios, including adaptive cruise and automatic obstacle avoidance systems for longitudinal acceleration and deceleration control, a curve path planning and lane changing model for lateral operation, etc. In early research, the main goal of these vehicle control strategies was to ensure vehicle safety. However, with the development of technology, scholars began to consider the comfort of passengers when designing vehicle control strategies. 

For mostly intelligent driving systems, such as adaptive cruise systems and automatic collision avoidance systems, the main method by which to improve passenger comfort is to reduce the maximum acceleration and jerk of the vehicle. To avoid the reduction of passenger comfort due to the frequent emergency braking of vehicles, Wang et al. [21] proposed an improved collision avoidance system based on vehicle communication, in which the deceleration of 4 m/s^2^ was set as the comfortable deceleration threshold. The simulation results revealed that the improved collision avoidance system enhanced the comfort of passengers while also ensuring safety. Lane-change trajectory planning is a key issue in intelligent driving technology; to improve passenger comfort during lane changing, some scholars have limited the maximum lateral acceleration and jerk of vehicles. Lee and Litkouhi [22] considered the influence of vehicle lateral motion on passenger comfort, and proposed a more flexible lateral control algorithm based on the relaxation algorithm, which can adjust the threshold of lateral acceleration in the control algorithm according to different levels of comfort requirements and generate different levels of smooth lane-change trajectories. However, in the design of the algorithm, they set the universal acceleration limit to 0.15 g, without considering the difference in the acceptability of different passengers for vehicle motion parameters. Heil et al. [23] also used acceleration and jerk as evaluation indexes of comfort, and established a lane-change trajectory planning model via a quintic polynomial. The model can generate a non-symmetrical anthropomorphic lane-change trajectory, which can improve passenger comfort. To improve passenger comfort when autonomous vehicles drive on curved sections of road, Artunedo et al. [24] planned smooth paths by using the OpenStreetMap open-source navigation data structure and converted the original navigation data into drivable space, which was then combined with the MINLP optimization algorithm. The algorithm considers the lateral acceleration and jerk of the vehicle in the loss function, and ultimately generates a smooth path to ensure passenger comfort. Considering the riding comfort of buses, when designing a vehicle control strategy of intelligent buses for curved road sections, Villagra et al. [25] considered not only the lateral acceleration and jerk of the vehicle but also the maximum longitudinal acceleration and the longitudinal acceleration range of the vehicle.

Through the above literature review, we can find that most of the intelligent control strategies considering passenger comfort, whether it is the lateral movement or the longitudinal motion of the vehicle, took vehicle acceleration and jerk as comfort indicators. However, when setting the index threshold, the difference in the acceptability of different passengers to vehicle motion parameters was not considered. In addition, to the best of our knowledge, we hardly found practical predictive models. In this study, we carried out a real riding experiment to simulate the typical driving state of a future autonomous vehicle, and collected the vehicle motion parameters under different conditions. In addition, we designed real-time collection equipment for subjective passenger comfort to collect passenger comfort. Based on the analysis of passenger comfort factors and differences in the acceptance of vehicle motion parameters by different passengers, a passenger comfort prediction model was established, which can help autonomous vehicle path planning and control strategy design.

## 2. Materials and Methods

The main research purpose of this article is to explore the impacts of the vehicle motion state, vehicle operating parameters, and passengers’ physiological characteristics on passenger comfort and then establish a passenger comfort prediction model so as to improve the comfort of autonomous vehicles. Experiments conducted with autonomous vehicles require the specific writing of corresponding algorithms to meet the needs of different vehicle motion states, and the overall operation is difficult. It is a common method to study passenger comfort when using a human driver to drive a traditional vehicle to simulate an autonomous vehicle, and the test results can provide a reference for the motion parameters of an autonomous vehicle [26]. Moreover, the anthropomorphism of autonomous vehicle control is the future development direction of autonomous vehicles. Therefore, research on the ride comfort of vehicles based on traditional drivers can provide a powerful reference for improving the algorithm design of the ride comfort of autonomous vehicles. 

### 2.1. Experimental Equipment

#### 2.1.1. Vehicle Motion Parameter Collection Equipment

A passenger car was selected as the research object, and the vehicle used was a Volkswagen Langyi (Xi’an, CN). The shift impact of manual transmission vehicles will affect the comfort of passengers [10], so an automatic transmission vehicle was selected. 

**Figure 1:**
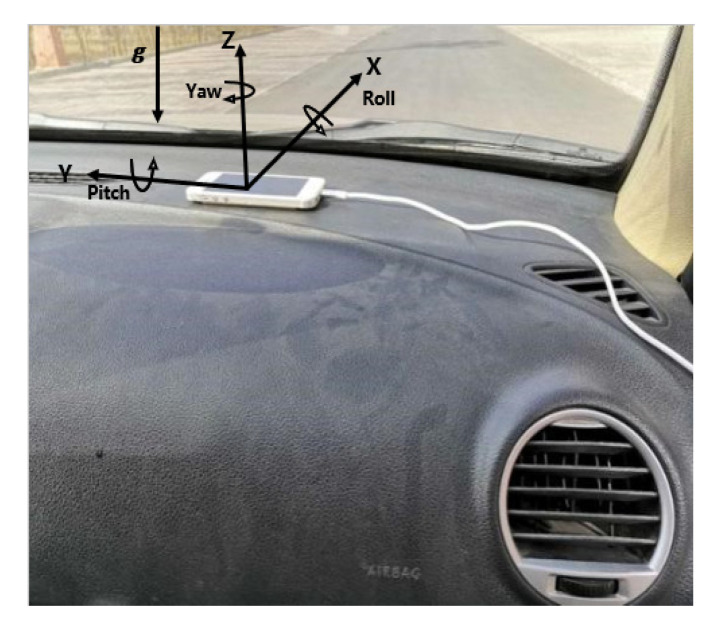
Illustration of the fixed location and axial diagram of the IOS smart device.

It is a common measure to monitor vehicle motion data by smartphone, and the accuracy of data collected by smartphone is consistent with that of high-precision gyroscope [27]. An iOS smart device used to collect data on the vehicle motion state in real-time is an iPhone 6S plus smartphone with running iOS 12.4. The built-in sensor of the mobile phone is an InvenSense MP67B, with a full-grid sensing range of the accelerometer of ±2 g, ±4 g, ±8 g, and ±16 g, and a full-grid sensing range of the gyroscope of ±250, ±500, ±1000, ±2000°/s. The position of the iOS device was fixed under the condition that the axis of iOS equipment was consistent with that of vehicle. The fixed position and axial diagram of the iOS smart device is depicted in Figure 1.

**Figure 2:**
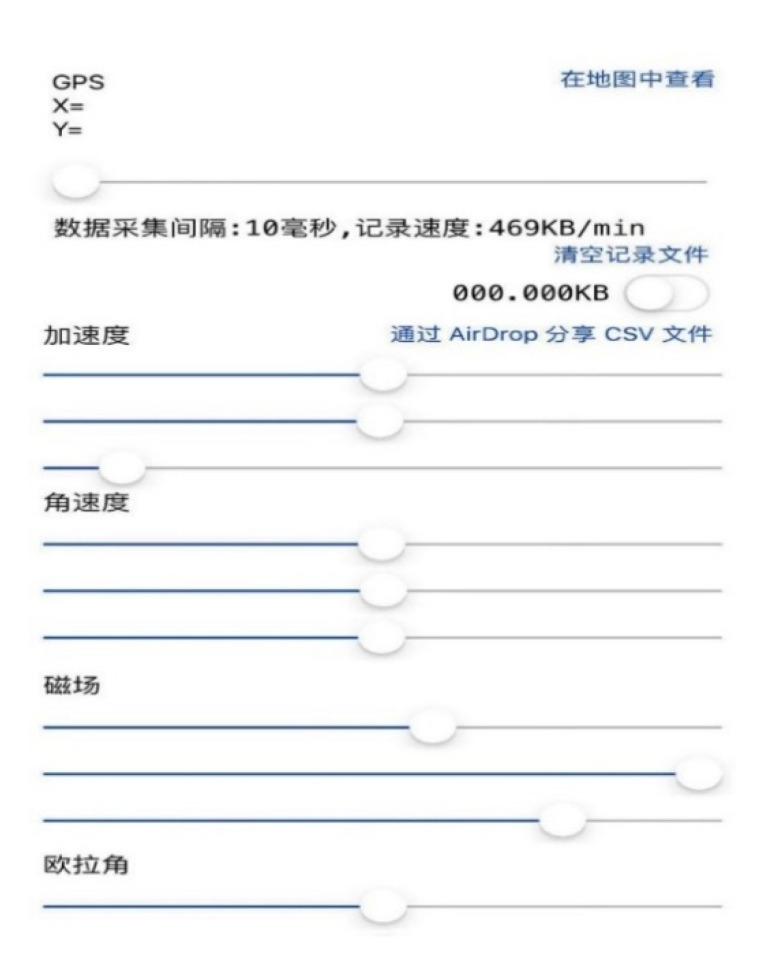
The user interface of the acceleration software (no English version, “加速度” means “acceleration”).

Acceleration software was used to collect the motion parameters of vehicles and objects in real time. The sampling frequency was set to 100 Hz. The user interface of the acceleration software is shown in Figure 2. 

#### 2.1.2. Real-Time Collection Equipment for Subjective Passenger Comfort

**Figure 3:**
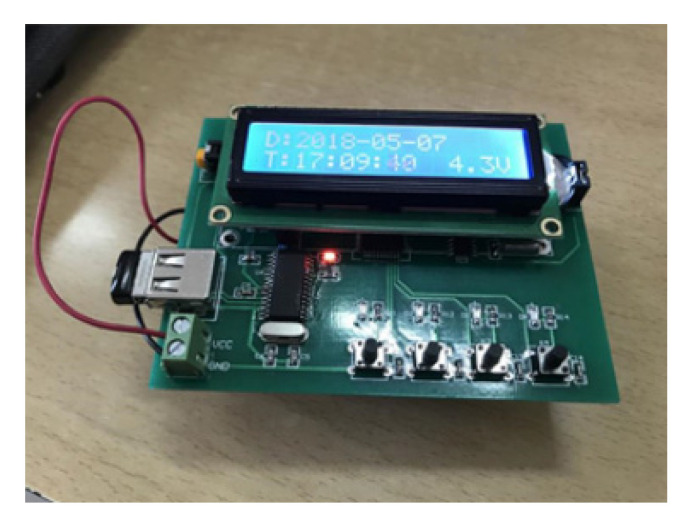
Illustration of the device for the collection of data on the subjective evaluation of passenger comfort.

In this experiment, to obtain real-time data on the passenger comfort perception, a device for the acquisition of the subjective evaluation results of passenger comfort was specially designed and developed and was used to collect related data under different vehicle motion states. The equipment was made by designing a relevant circuit diagram, writing a program, and importing it into a K60 single-chip microcomputer, which was then welded to a circuit board, as depicted in Figure 3. The four different buttons in the device were denoted as #1, #2, #3, and #4, which respectively corresponded to the perceptions of no discomfort, discomfort, no motion sickness, and motion sickness. In the experiment, the passengers were instructed to record their current riding experience by pressing the corresponding button, which was convenient for the later data statistics. 

#### 2.1.3. Communication Equipment

According to the test requirements, the driver drove the vehicle to complete multiple driving operations, including acceleration, deceleration, and turning at different speeds and accelerations. In addition, after a single driving operation, the driver prompted the participants to make a ride comfort evaluation. To prevent the driver from working under too much pressure, a smartphone was placed on the experimental vehicle to realize the real-time communication between the driver and the external staff, during which the driver was instructed on which operation to perform. 

### 2.2. Participants

**Figure 4:**
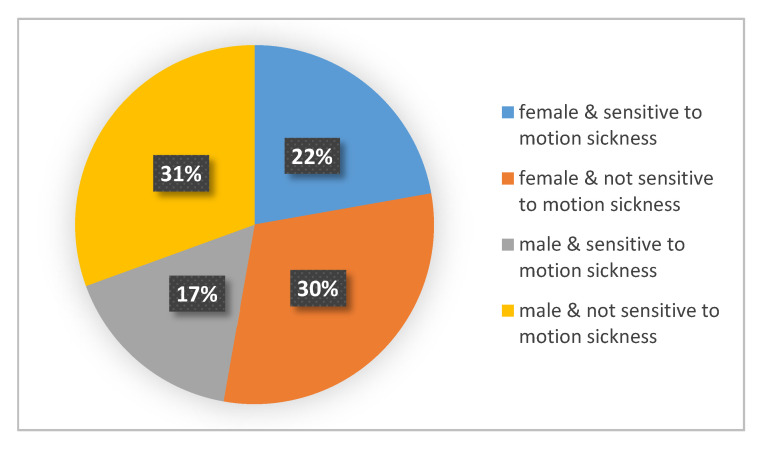
Illustration of different characteristics of the subjects.

The volunteer participants in this experiment were students from Chang’an University, Xi’an, China, and they were recruited by posting information on social media platforms. Participants were initially screened based on the basic information (including gender, age, self-assessment of motion sickness susceptibility, etc.) filled by volunteers, and then they were asked to complete the Motion sickness susceptibility questionnaire (MSSQ)- [28]. According to the evaluation of the motion sickness susceptibility questionnaire, the subjects were divided into two categories: sensitive to motion sickness and not sensitive to motion sickness. In total, 36 participants (19 female, 17 male) took part in the study. The participants’ ages ranged from 21 to 66 years (mean = 24.3 yrs., SD = 3.8 yrs.). The proportions of subjects with different motion sickness susceptibilities and genders are illustrated in Figure 4.

### 2.3. Driver and Maneuvers

The driver selected for this test was a 36-year-old male driver with a driving experience of more than 13 years, a total driving mileage of more than 100,000 km, and no traffic accidents in the past five years. Before the formal test, the driver was trained for two days to fully understand the purpose of the test and to become familiar with the test vehicle and test process to ensure that the driving operation could meet the test requirements.

To best simulate the driving conditions of future intelligent vehicles during the daily driving process, the trained driver carried out driving operations that are common in urban road traffic, including acceleration, deceleration, and turning operations. Acceleration and deceleration involve the longitudinal movement of the vehicle, while turning involves the lateral movement of the vehicle. During the experiment, the driver was required to keep driving at a constant speed of 60 km/h except when performing longitudinal motion tasks. The process of the deceleration mode was that the driver conducted a braking operation when driving at a constant speed of 60 km/h until the vehicle stopped completely; the process of the acceleration mode was that the driver accelerated from a stationary condition to 60 km/h. There was a short break between the acceleration and deceleration conditions. The turning conditions are selected to be tested on the curved part of the circular test road in the automobile proving ground, because vehicles can easily slide and roll over when turning at high speeds, thereby threatening the safety of both the driver and passengers. Therefore, in the experiment, a low-speed uniform turn was selected, and the various turning speeds were 20, 30, and 40 km/h.

### 2.4. Experimental Site

**Figure 5:**
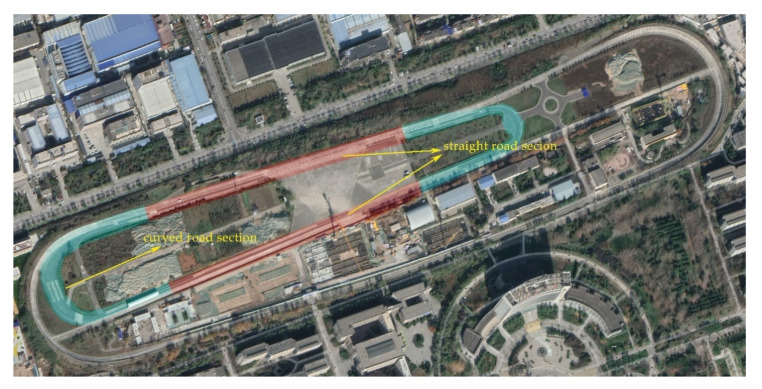
Image of the automobile performance testing ground at Chang’an University, which covers an area of 330,000 m^2^.

This experiment mainly aimed at the investigation of the three vehicle motion states of acceleration, deceleration, and turning to analyze the influence of the vehicle motion state on passengers’ riding comfort. The high-speed circular runway in the automobile performance testing ground at Chang’an University was used in the experiment. The runway is divided into different sections; the vehicle acceleration and deceleration conditions were completed in the straight-road section, while the vehicle turning condition was completed in the curved section. The test track and its division into sections are depicted in Figure 5.

### 2.5. Experimental Procedure

The subjects were instructed to fill in a questionnaire with their basic information and were then told the purpose of the test and were required to sign an informed consent form. Before the formal test, it was necessary to train the subjects to use the subjective ride comfort acquisition equipment and ensure that they could select the appropriate buttons according to their own comfort. To ensure that the timing of the iOS smart device was consistent with the timing of the passengers holding the subjective evaluation result collection device, the time of the iOS smart device was calibrated. After the calibration was completed, the iOS smart device was fixed horizontally on the vehicle to record data on the vehicle driving characteristics. In addition, when the equipment was fixed, the axial direction of the equipment was aligned with that of the vehicle as much as possible to reduce possible errors in data processing.

During the real vehicle experiment, the driver communicated with the external staff through the mobile phone communication terminal to receive instructions on the performance of different vehicle operations. At the beginning of a single operation, the driver verbally communicated the word “start”, and after the completion of a single operation, the driver verbally communicated the word “end” to direct the test crew to evaluate the riding comfort of the corresponding operation. Meanwhile, the off-site staff recorded the start and end times of the operation according to the commands for future data selection. In a single experimental run, three subjects were present in the vehicle at the same time. The entire process lasted for about 30 min, during which the driver completed nine acceleration operations, nine deceleration operations, and eighteen turning operations. After the test, each subject was given 100 yuan as compensation for their participation.

### 2.6. Data Processing

A total of 1296 groups of data were collected in this real-vehicle experiment, including real-time data on the vehicle motion state collected and stored by the IOS smart device, data on the passenger comfort evaluation collected by the related equipment, and basic information collected by the questionnaire.

**Figure 6:**
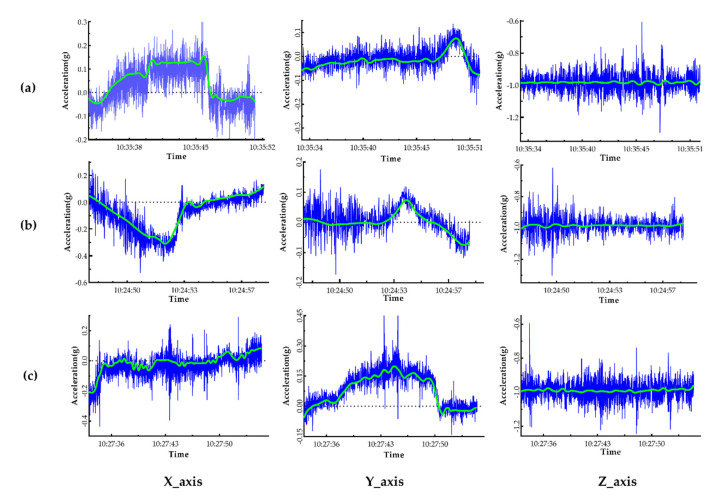
Illustration of the vehicle acceleration data before and after filtering for a certain experimental operation. The blue lines represent the original acceleration, and the green lines represent the filtered vehicle acceleration. (**a**) The acceleration during the acceleration operation; (**b**) the acceleration during the deceleration operation; (**c**) the acceleration during the turning operation.

Many parameters (such as three-axis acceleration, angular acceleration, Euler angle, and magnetic field) of the vehicle could be collected through the iOS equipment, but we only explored the impact of three-axis acceleration-related parameters on comfort. After the preliminary screening of the data, the acceleration data of each axis of the vehicle was obtained, and a simple analysis and drawing were carried out. Due to factors such as equipment and driving operation, the vehicle acceleration data graph exhibits obvious small-scale floating and “burr” phenomena; thus, the “db4” wavelet function in MATLAB was used to filter the data. Figure 6 presents the filtered vehicle acceleration data after a certain experimental operation—namely, the vehicle motion states of acceleration, deceleration, and turning.

From Figure 6, it can be seen that the changes in axial acceleration were different under the motion states of vehicle acceleration, deceleration, and turning. In the figure, the *X*-axis, *Y*-axis, and *Z*-axis, respectively, represent the longitudinal acceleration along the driving direction of the vehicle, the lateral acceleration perpendicular to the driving direction in the horizontal plane, and the vertical acceleration. During the acceleration and deceleration operations, the acceleration of the *X*-axis in the vehicle traveling direction, that is, the longitudinal acceleration, was found to changes significantly. During a turning operation, the *Y*-axis acceleration, that is, the lateral acceleration, was found to change significantly [29]. Therefore, in consideration of the actual driving situation, the main focuses of further investigation were the influences of the longitudinal acceleration, jerk, and duration of a single operation on passenger comfort under the vehicle acceleration and deceleration conditions and the influences of the lateral acceleration, jerk, and duration of a single operation on passenger comfort under the vehicle turning condition. 

## 3. Results

### 3.1. Reliability Test of Subjective Comfort Evaluation of Subjects

To determine the relationship between the vehicle acceleration amplitude and passenger comfort, the MATLAB filter toolbox was used to extract the longitudinal acceleration amplitude under the acceleration and deceleration operations and the lateral acceleration amplitude under the turning operation [30,31,32]. If the ride comfort of the subjects was not related to the acceleration amplitude, which is contrary to the results of previous research, it was considered that the subjective evaluation of the test subjects had lower reliability; otherwise, it had higher reliability.

**Figure 7:**
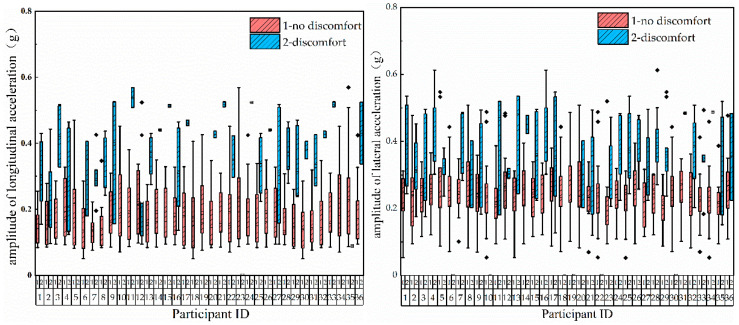
Box diagrams of the vehicle acceleration amplitude and passenger comfort evaluation results.

The longitudinal acceleration data amplitudes under nine acceleration operations and nine deceleration operations, and the lateral acceleration data amplitudes under 18 turning operations, were respectively extracted. Combined with the subjective evaluation results of riding comfort of the 36 subjects, the related box diagram is presented in Figure 7.

It can be seen from Figure 7 that, for the same passenger, the acceleration amplitude range corresponding to discomfort is larger than that of comfort, or discomfort is slightly crossed with comfort, but the former is closer to the upper part of the longitudinal (lateral) axis. Therefore, it can be considered that the greater the amplitude of the lateral and longitudinal acceleration of the vehicle, the more likely it is to cause passenger discomfort, which is in line with common sense. The subjective evaluation results of the tested passengers in this real-vehicle test therefore have certain credibility and can be used for subsequent research on passenger comfort.

### 3.2. The Relationships between the Evaluation Results of Passenger Comfort and Their Physiological Characteristics and Vehicle Operating Parameters

The subjective evaluation results of the passengers were divided into the discomfort group and the no discomfort group, and the passengers’ gender and susceptibility to motion sickness, the duration of a single vehicle operation, the acceleration, and the jerk were used as the independent variables to investigate their effects on the subjective evaluation results under three motion states. To analyze the factors of the passengers’ physiological characteristics and vehicle operating parameters, the single-factor analysis method was used to test the statistically significant factors between the two groups. Taking passenger comfort as the dependent variable, using the t test method for passenger sex and motion sickness susceptibility data, using the $\chi^{2}$ test method for the single operation time, acceleration, and jerk of the vehicle, to perform one-way analysis of variance, where the test level has been set to 0.05. Significant factors were then introduced into the binary logistic regression analysis model to explore the relationships between the passengers’ evaluation results and their physiological characteristics and vehicle operating parameters.

For the acceleration state, it was found that the passenger’s susceptibility to motion sickness, vehicle acceleration, and jerk were correlated with passenger comfort. No correlation was found between passenger comfort and the passenger’s gender, nor between passenger comfort and the length of a single vehicle operation. In view of relevant research showing that gender is an important factor that affects the passenger experience [19], the motion sickness susceptibility of the passenger, the passenger’s gender, and the vehicle acceleration and jerk were introduced into the binary logistic regression analysis model to further determine the factors that affect passenger comfort. The regression analysis results are reported in Table 1. The passenger’s susceptibility to motion sickness and vehicle acceleration were entered into the regression equation of the passenger comfort evaluation results. The results show that, compared with the passengers susceptible to motion sickness, the passengers less susceptible to motion sickness indicated experiencing 21% less discomfort. In other words, the passengers who were susceptible to motion sickness were more likely to indicate discomfort than those who were not susceptible to motion sickness; moreover, the greater the acceleration of the vehicle, the more likely it was to cause passenger discomfort. Other independent variables, such as the gender of the passenger, the duration of a single vehicle operation, and the correlation between the jerk and the evaluation results, were not found to be statistically significant.

Similarly, during the deceleration state, the passenger’s susceptibility to motion sickness, the duration of a single operation, and the acceleration of the vehicle were found to be important factors related to the evaluation results of passenger comfort. The passengers who were susceptible to motion sickness were more likely to indicate discomfort than those who were not susceptible to motion sickness. The vehicle acceleration was found to be positively correlated with the evaluation results, indicating that the greater the vehicle acceleration, the more likely it was to cause passenger discomfort. 

During the turning state, the passenger’s gender and vehicle acceleration were found to be important factors correlated with the evaluation results of passenger comfort. Female passengers were more likely to indicate discomfort than male passengers, while vehicle acceleration was found to be positively correlated with the passenger comfort evaluation results, indicating that the greater the vehicle acceleration, the more likely it is to cause passenger discomfort.

### 3.3. Analysis of the Difference in Passengers’ Acceptance of the Vehicle Motion State Parameters

The relevant factors that affect the evaluation of passenger comfort were determined via binary logistic regression analysis. To further study the effects of the interactions between the passenger’s physiological characteristics, the vehicle motion state, and the vehicle operating parameters on passenger comfort, the differences between the acceptance to the same vehicle operating parameters by passengers with different physiological characteristics were explored. In this study, the physiological characteristics of the passengers included their gender and motion sickness susceptibility; the vehicle motion states included the acceleration, deceleration, and turning operations; and the vehicle operating parameters included vehicle acceleration, jerk, and the duration of a single operation.

**Figure 8:**
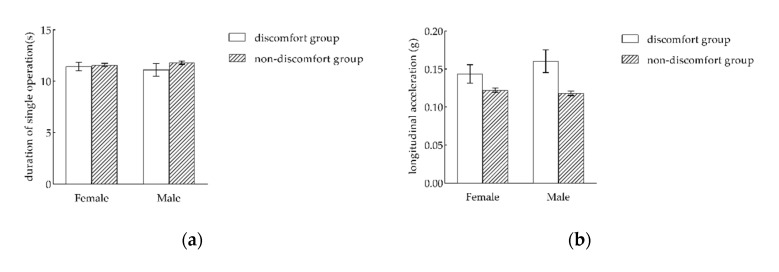

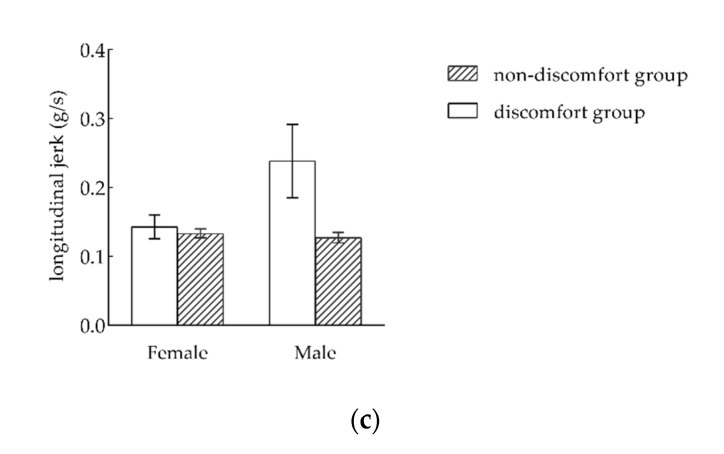
The differences in the acceptance of various vehicle operation parameters by passengers with different genders. (**a**) The duration of a single operation; (**b**) the longitudinal acceleration; (**c**) the longitudinal jerk.

Taking the difference analysis of the acceptance of the same vehicle operating parameters by passengers with different genders under the acceleration operation as an example, a *t*-test was conducted to analyze the differences in discomfort between men and women. The results of the *t*-test are presented in Figure 8. 

As presented in Figure 8a, there was no significant difference (*p* = 0.646 > 0.05) in the duration of a single operation between the female discomfort group (μ = 11.447, σ = 0.415) and the male discomfort group (μ = 11.113. σ = 0.615). Additionally, there was no significant difference (*p* = 0.344 > 0.05) in the duration of a single operation between the female non-discomfort group (μ = 11.595, σ = 0.158) and the male non-discomfort group (μ = 11.816, σ = 0.172). Therefore, there was no significant difference in the acceptance of the duration of a single operation between male and female passengers.

As presented in Figure 8b, there was no significant difference (*p* = 0.397 > 0.05) in the vehicle acceleration between the female discomfort group (μ = 0.144, σ = 0.012) and the male discomfort group (μ = 0.160, σ = 0.015). Additionally, there was no significant difference (*p* = 0.334 > 0.05) in the vehicle acceleration between the female non-discomfort group (μ = 0.122, σ = 0.003) and male non-discomfort group (μ = 0.118, σ = 0.003). Therefore, there was no significant difference in the acceptance of vehicle acceleration between male and female passengers.

As presented in Figure 8c, the vehicle jerk of the female discomfort group (μ = 0.143, σ = 0.017) was significantly less (*p* = 0.049 < 0.05) than that of the male discomfort group (μ = 0.238, σ = 0.053). Additionally, there was no significant difference (*p* = 0.547 > 0.05) in the vehicle acceleration speed between the female non-discomfort group (μ = 0.133, σ = 0.007) and the male non-discomfort group (μ = 0.127, σ = 0.008). Therefore, under the comprehensive consideration of these findings, there was no significant difference in the acceptance of vehicle jerk between male and female passengers.

Similarly, no significant difference was found between passengers with different susceptibilities to motion sickness regarding the different vehicle operating parameters during the acceleration operation. In contrast, during the deceleration operation, there were significant differences in the acceptance of vehicle acceleration between male and female passengers, and the acceptance of acceleration by female passengers was significantly less than that by male passengers. There were also significant differences in the acceptance of vehicle acceleration between passengers with different susceptibilities to motion sickness; those who were susceptible to motion sickness were significantly less accepting of vehicle acceleration than those who were not susceptible to motion sickness. Regarding the turning operation, no significant difference was found in the acceptance of the operating parameters of the vehicle by male and female passengers, nor in the acceptance of the operating parameters by passengers with different susceptibilities to motion sickness.

## 4. Passenger Comfort Evaluation and Prediction Method Based on BiLSTM

In order to overcome the short-term memory distance and gradient explosion of the recurrent neural network (RNN), in 1997, Hochreiter and Schmidhuber [33] first proposed the long short-term memory network (LSTM), a kind of time recurrent neural network. It enables the network to merge the output result of the previous moment with the new input result of the current moment, together as the output of the current moment. There are two LSTM layers in the bidirectional long and short-term memory network structure, which can simultaneously utilize the information of the past and future moments. As the advantages of the bidirectional long and short-term memory network model have been discovered by experts and scholars, the model has been well applied in many time series tasks and achieved good results. In 2005, Graves et al. [34] first applied the bidirectional LSTM neural network model to classification questions and achieved better results than the unidirectional LSTM model. 

A passenger comfort prediction model was established based on the bidirectional long short-term memory (BiLSTM) network, which was then used to predict the ride comfort of passengers by combining the relationships between relevant influencing factors and passenger comfort evaluation.

### 4.1. Model Input Parameters

The model test data set was composed of 324 sets of vehicle acceleration data, 324 sets of vehicle deceleration data, and 648 sets of vehicle turning data obtained from the real-vehicle experiment. There were eight basic variables in each group of data, among which the input variables included the vehicle motion state parameters (acceleration operation is recorded as 1, deceleration operation is recorded as 2, turning operation is recorded as 3), longitudinal acceleration, lateral acceleration, longitudinal jerk, lateral jerk, the passenger’s gender (female is recorded as 0, male is recorded as 1), and the passenger’s susceptibility to motion sickness (passengers who are susceptible to motion sickness are recorded as 0, passengers who are not susceptible to motion sickness are recorded as 1). The output variable of the model is the passenger comfort evaluation result (0 indicates no discomfort, 1 indicates discomfort).

As was determined in Section 3, differences were found in the subjective evaluation results of passenger comfort under different vehicle motion states. The vehicle acceleration, passenger’s gender, and passenger’s susceptibility to motion sickness are also important factors that affect passenger comfort. The preceding analysis did not discover a correlation between vehicle jerk and passenger comfort; however, because relevant research shows that a certain relationship exists [18,35,36], vehicle jerk was also used as an input variable in the passenger comfort prediction model. To further evaluate the impact of the vehicle operating parameters on the passenger comfort prediction model, the combinations of input variables are divided into three categories—A, B, and C—as listed in Table 2. 

The prediction effects of the passenger comfort model of the three input variable combinations A, B, and C were compared and analyzed, and the parameter combination X with the best prediction effect was determined when only the vehicle operating state parameters were considered. After including the passenger’s gender classification and motion sickness susceptibility classification, the input variable combination D was developed as the final input parameters of the passenger comfort prediction model.

### 4.2. Model Evaluation Indicators

The prediction accuracy rate of the passenger evaluation results is used as the first-level measurement standard of the BiLSTM model. The accuracy rate (Accuracy) refers to the proportion of samples for which the model makes correct predictions; in this article, the accuracy rate is the ratio of the number of correctly predicted passenger evaluation results out of the total number of samples. If the model prediction accuracy rate cannot be used to accurately measure the prediction results, the F1-score index (the F1 value is a parameter used to measure the accuracy of model testing) of the model is further selected as the secondary measurement standard of the model.

Accuracy can be directly determined from the accuracy rate curve of the model prediction results. The F1-score index is calculated by the Precision and Recall of the model prediction. In this article, the precision of the model refers to the proportion of samples for which the model predictions are correct among all the results that the model predicts as discomfort, and the recall rate of the model refers to the proportion of samples for which the model predictions are correct among all the results whose true value is discomfort. The F1 value is the harmonic average of the precision and recall of the model prediction; its value range is [0, 1], and the F1 value is optimal when it is equal to 1. With the decrease of the F1 value, the model prediction effect decreases, and the effect is the worst when the value is equal to 0.

From the confusion matrix, the four values of true positives (TP), false positives (FP), false negatives (FN), and true negatives (TN) of the model can be obtained. In this article, TP refers to the samples whose real values were comfort and were predicted to be comfort. FN refers to the samples whose real values were comfort but were predicted to be discomfort. From these four values, the Precision, Recall, and F1 score of the model can be calculated via the following equations:

Precision = TP/(TP + FP)(1)

Recall = TP/(TP + FN)(2)

F1 Score = 2 * Precision * Recall/(Precision + Recall)(3)

### 4.3. Model Prediction Results

The acceleration, deceleration, and turning data were integrated to form the model test data set. 80% of the statistical data set was used as the training set to train the model, while 20% of the data set was used to test the model. The test set included a total of 207 sets of data, of which there were 104 sets of participants without discomfort and 103 sets of participants with discomfort.

**Figure 9:**
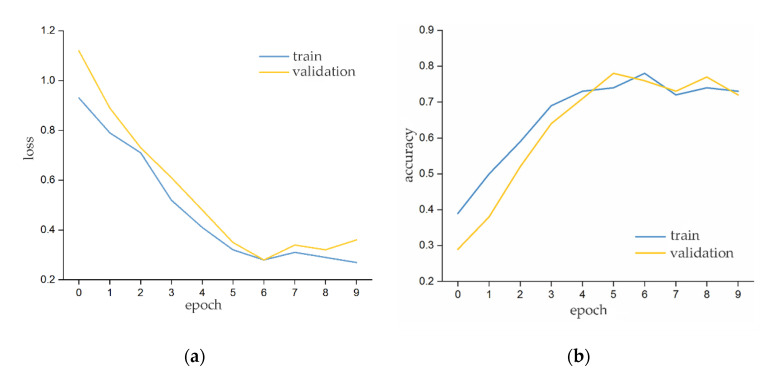
The (**a**) loss value curves and (**b**) accuracy curves of the model with the input parameter combination A.

The training set and test set data of parameter combination A were imported into the comfort prediction network for model training and prediction. Figure 9a presents the changes in the model loss values of the training set and test set with the number of epochs, and Figure 9b presents the changes in the model prediction accuracy of the training set and test set with the number of epochs.

**Figure 10:**
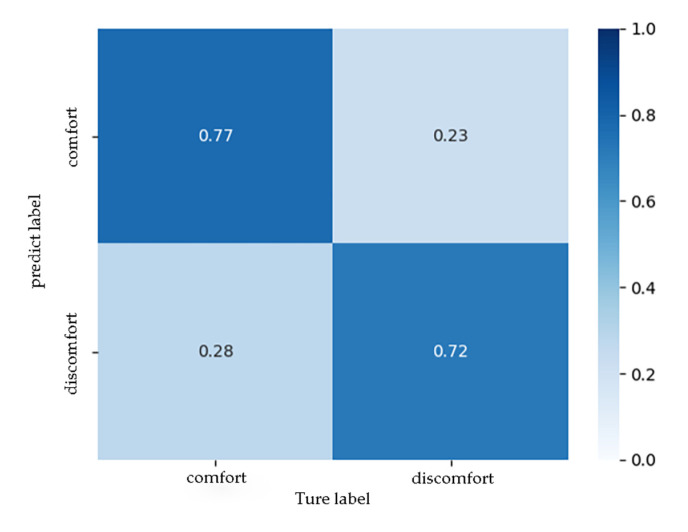
The confusion matrix of the test set when the parameter combination A was input.

According to Figure 9, considering the results of the training set and the test set, the training was stopped after the sixth epoch. Figure 10 presents the confusion matrix of the test set, from which it can be seen that, when the parameter combination A was input, the model correctly identified 72% of the participants’ discomfort states and 77% of the non-discomfort states. The overall accuracy of the model was 75%, and the prediction effect was general.

Similarly, when the parameter combination B was input, the model correctly identified 64% of the passengers’ discomfort states and 72% of the non-discomfort states. The overall accuracy of the model was 69%, and the prediction effect was poor. When the parameter combination C was input, the model correctly identified 76% of the passengers’ discomfort states and 81% of the non-discomfort states. The overall accuracy of the model was 79%, and the prediction effect was good.

From the model prediction results of the input parameter combinations A, B, and C, it is evident that when parameter combinations A and C were input, the model prediction accuracy rate was significantly better than that when parameter combination B was input. To further determine the prediction effect of the model with the input parameter combinations A and C, the F1-score index values of the prediction results based on the BiLSTM model under the three input parameter combinations were calculated, and are reported in Table 3.

From Table 3, it can be found that the F1 score of the model under the input parameter combination C was better than that under the input parameter combination A; thus, the prediction effect of the model was better under the input parameter combination C. This is because, when the acceleration data and the jerk data of the vehicle are considered comprehensively, the predictive effect of the model is better than that when only the vehicle acceleration or only the jerk of the vehicle is considered.

**Figure 11:**
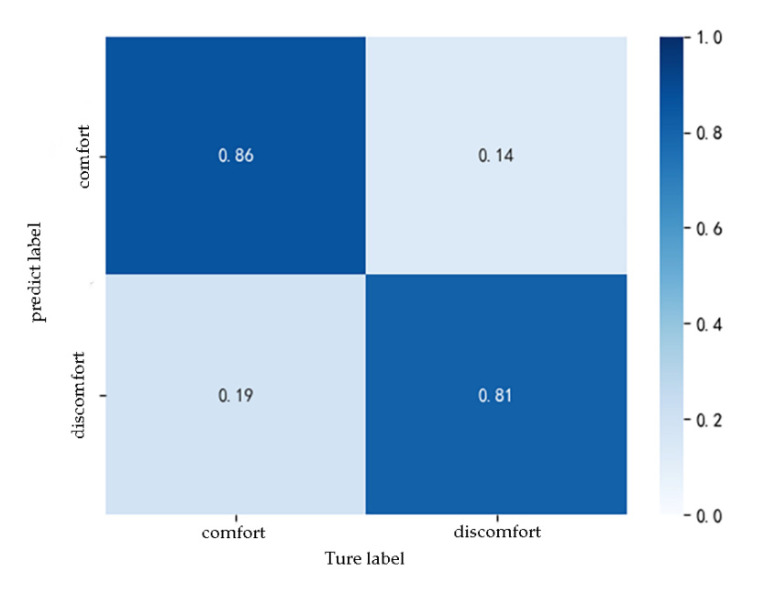
The confusion matrix of the test set when parameter combination D was input.

Because the analysis in Section 3 of this article revealed that the passenger’s gender and susceptibility to motion sickness are related to the passenger’s comfort evaluation results, the passenger’s physiological characteristic indicators were added to parameter combination C to form a new parameter combination D. Figure 11 presents the confusion matrix of the test set when the parameter combination D was input. The model correctly identified 81% of the passengers’ discomfort states and 86% of the non-discomfort states. The overall prediction accuracy of the model was 84%. Via Equations (1)–(3), the precision of the model was calculated to be 81.6%, and the recall was 86.4%. This demonstrates that the model can better predict passenger comfort. In addition, the F1 score of the model was 0.84, indicating that the output results of the model were credible.

In summary, it can be found that when only the vehicle acceleration was considered (parameter combination A), the accuracy of the model was only 75%, which is higher than that only considering the jerk of the vehicle (parameter combination B). When both acceleration and jerk were considered (parameter combination C), the prediction accuracy of the model increases to 79%. When the passenger characteristics were further considered (parameter combination D), the model’s prediction accuracy was up to 84%. By comparing the prediction effect of the model under different combinations of input parameters, it is further proved that the comfort of passengers was affected by vehicle acceleration, jerk and personal characteristics of passengers, and the impact of vehicle acceleration is the greatest. When the acceleration, jerk and personal characteristics of passengers were input into the model, the prediction accuracy is high, and the subjective comfort of passengers can be judged according to the vehicle motion parameters, which can be used for the control decision of intelligent vehicles.

## 5. Conclusions

The original purpose of vehicle intelligent control algorithms and motion models is to ensure vehicle safety. However, with the maturity of the control algorithm and model, more and more scholars will pay more attention to passengers’ comfort, driver’s driving habits, energy conservation, and environmental protection when studying control algorithm and model. Among them, comfort is an important factor in the design of smart cars. Comfortable autonomous cars will increase people’s acceptance of smart cars and will further increase the penetration rate of intelligent cars. However, unlike traditional car comfort research, which mainly considers the impact of vehicle chassis vibration on passengers, the comfort of intelligent cars needs to further consider the impact of vehicle control algorithms on passenger comfort. The research on passenger comfort is helpful to understand the mechanism of affecting passengers’ subjective comfort, improve the theory of motion sickness, and provide a theoretical basis for the research of intelligent vehicle automatic driving control algorithm and decision making and the design of human–computer interaction systems.

In the present study, a human-driven experiment in an automobile test ground was carried out to simulate the typical driving state of a future autonomous vehicle during the process of vehicle motion. In the experiment, vehicle motion parameters and the comfort evaluation results of passengers with different physiological characteristics were collected. A single-factor analysis method and binary logistic regression analysis model were used to determine the factors that affect the evaluation results of passenger comfort. It was found that the vehicle acceleration and susceptibility to carsickness are related to passenger comfort under longitudinal conditions. During the lateral operation, the gender and vehicle acceleration are related to passenger comfort under longitudinal conditions. The differences in the acceptance of vehicle operating parameters by passengers with different physiological characteristics are explored, which helps to lay the foundation for the personalized algorithm design of unmanned vehicles. In addition, through regression analysis, we found that vehicle acceleration is the factor that has the greatest impact on passenger comfort. Therefore, acceleration should be given priority in the control algorithm design of autonomous vehicle. According to our research results, we suggest establishing a multi-objective optimization function in local trajectory planning of intelligent vehicles, including vehicle acceleration, passenger gender, carsickness susceptibility, and other indicators, and using algorithms such as fuzzy logic controller to allocate the weight function in the multi-objective function so as to improve the comfort of passengers on the premise of ensuring safety.

In order to further assist the motion operation planning of intelligent vehicle, a passenger comfort prediction model based on BiLSTM was established. The model can predict the comfort of passengers according to the physiological characteristics of passengers and the motion parameters in vehicle planning. The vehicle longitudinal acceleration, lateral acceleration, longitudinal jerk, lateral jerk, vehicle motion state classification, passenger’s gender, and passenger’s susceptibility to motion sickness were used as model input variables, and the accuracy of the passenger comfort prediction model was found to reach 84%, which can be applied to the control decision of intelligent vehicle and improve the riding comfort of passengers. 

## 6. Limitations and Future Works

In order to improve the comfort of autonomous vehicles, a real riding experiment was carried out to simulate the typical driving state of a future autonomous vehicle during the process of vehicle motion. Through the self-designed passenger comfort real-time acquisition equipment, the passengers’ real-time evaluation of vehicle driving comfort was realized. According to the results of passenger comfort evaluation, the effects of vehicle motion parameters and passenger physiological characteristics on comfort under different motion conditions were analyzed, and the differences in passenger acceptance of vehicle operating parameters were explored. Finally, a passenger comfort prediction model was established, which provides the basis for the control algorithm design of autonomous vehicles. However, due to the limitation of experimental conditions and other factors in this study, only 36 subjects were recruited, and the amount of data was not large enough. In addition, we did not pay attention to the changes in the numerical physiological characteristics of passengers during the ride, such as posture changes. Follow-up research can be carried out from the following aspects:

(1) Increasing the number of subjects to validate the reliability of our model;
(2) Increasing the number of motion conditions, such as lane changing or overtaking, to explore the comfort of passengers under these typical conditions;
(3) Carrying out autonomous vehicle trials to explore the comfort of autonomous vehicles in the future;
(4) Considering the difference of passenger posture changes under different vehicle motion states and exploring the influence of this factor on passenger comfort evaluation.

## Figures and Tables

**Table 1:** Binary logistic regression analysis of vehicle acceleration operation and subjective comfort evaluation results.

Factor	B	S.E.	Walds	Sig.	Exp(B)	Exp(B) 95% Confidence Interval	
						Lower Limit	Upper Limit
Acceleration	16.963	4.814	12.416	0.000	2.3 × 10^7^	1.9 × 10^3^	2.9 × 10^11^
Motion sickness susceptibility	−1.563	0.481	10.564	0.001	0.210	0.082	0.538
Constant	−4.077	0.724	31.715	0.000	0.017		

*p* < 0.050 indicates statistical significance. B represents the regression coefficient. S.E. represents the standard error. Sig. represents the significance.

**Table 2:** Input variables of the passenger comfort model considering the vehicle operation state parameters.

Category	Input Variables
A	vehicle motion state, longitudinal acceleration, lateral acceleration
B	vehicle motion state, longitudinal jerk, lateral jerk
C	vehicle motion state, longitudinal acceleration, lateral acceleration, longitudinal jerk, lateral jerk

**Table 3:** Related indexes of the model prediction results under the inputs of parameter combinations A, B, and C.

Input Variables	Precision	Recall	F1 Score
Parameter combination A	76.7%	73.1%	0.75
Parameter combination B	71.8%	66.7%	0.69
Parameter combination C	80.6%	76.9%	0.79
